# Construction of an intercultural care program for international migrants in northwestern Mexico

**DOI:** 10.1590/1980-220X-REEUSP-2023-0024en

**Published:** 2023-09-25

**Authors:** Abraham Isaac Esquivel Rubio, Claudia Jennifer Domínguez Chávez, Laura Alicia Velarde Valenzuela

**Affiliations:** 1 Universidad Autónoma de Baja California, Facultad de Enfermería, Mexicali, Baja California, México.

**Keywords:** Emigrants and Immigrants, Culturally Competent Care, Cultural Competency, Nursing Care, Emigrantes e Imigrantes, Assistência à Saúde Culturalmente Competente, Competência Cultural, Cuidados de Enfermagem, Emigrantes e Inmigrantes, Asistencia Sanitaria Culturalmente Competente, Competencia Cultural, Atención de Enfermería

## Abstract

**Objective:**

To describe the construction process of an intercultural care program for international migrants in northwestern Mexico.

**Method:**

Report of professional experiences, according to what was suggested by Daltro and Faria.

**Results:**

The development and evolution of care for international migrants has favored the elaboration of a community-like Social Service Program for students of a public university in northwestern Mexico, so that intercultural health and health care for this population become part of the curricular training of the new generations of nursing graduates, in a context in which international migration is a topic of great social and cultural relevance.

**Conclusion:**

The construction and application of the *Salud-Migrante* program will enhance compliance with international recommendations on universal health for migrants, promoting respect for identity and cultural diversity in their actions.

## RESUMEN

**Objetivo:**

Describir el proceso de construcción de un programa de cuidado intercultural a personas migrantes internacionales en el noroeste de México.

**Método:**

Relato de experiencias profesionales, acorde a lo sugerido por Daltro y Faria.

**Resultados:**

El desarrollo y evolución del cuidado a personas migrantes internacionales ha favorecido la estructuración de un Programa de Servicio Social de tipo comunitario para estudiantes de una universidad pública en el noroeste de México, por lo que la salud intercultural y el cuidado a la salud a esta población se vuelve parte en la formación curricular de las nuevas generaciones de licenciados en enfermería, en un contexto en que la migración internacional es tema de gran relevancia social y cultural.

**Conclusión:**

La construcción y aplicación del programa Salud-Migrante fortalecerán el cumplimiento de las recomendaciones internacionales en materia de salud universal para personas migrantes, promoviendo en sus acciones el respeto a la identidad y diversidad cultural.

## RESUMO

**Objetivo:**

Descrever o processo de construção de um programa de atendimento intercultural para migrantes internacionais no noroeste do México.

**Método:**

Relato de experiências profissionais, conforme sugerido por Daltro e Faria.

**Resultados:**

O desenvolvimento e a evolução do atendimento aos migrantes internacionais favoreceu a estruturação de um Programa de Serviço Social de tipo comunitário para estudantes de uma universidade pública do noroeste do México, de modo que a saúde intercultural e a atenção à saúde dessa população tornem-se parte da formação curricular do novas gerações de graduados em enfermagem, em um contexto em que a migração internacional é um tema de grande relevância social e cultural.

**Conclusão:**

A construção e aplicação do programa Salud-Migrante fortalecerá o cumprimento das recomendações internacionais sobre saúde universal para migrantes, promovendo o respeito à identidade e à diversidade cultural em suas ações.

## INTRODUCTION

Recent estimates indicate that 3.6% of the global population are international migrants, the equivalent of 281 million people^(1)^ which are recognized worldwide as vulnerable populations in what regards health problems, derived from the conditions in which their transit to the destination country occurs. Among these, not having access to health services, violence in the migratory process, socioeconomic barriers, illiteracy, language differences, and even the development of risk behaviors, driven by the epidemiological conditions of the countries through which they transit, can be considered^(2,3,4)^.

On this basis, it should be considered that the presence of migrants in a particular context implies the existence of cultural diversity, since there are many nationalities, languages, habits, beliefs, and practices that move with them^(5)^. Under these settings and characteristics, one can state that this population is at risk of developing and acquiring communicable and non-communicable diseases, accidents, addiction and mental health problems, among others. This has led nursing, as a care science, to generate theoretical-methodological contributions that seek to understand, study, and address these social problems in a culturally sensitive way^(6,7,8)^.

Regarding the existing relationship between migrants and new contexts, it becomes necessary to consider the term of interculturality, which is defined as, “a communication and interaction tool that focuses on creating the conditions for dialogue, conflict management, and negotiation between individuals in diverse societies”^(9)^. Under this premise, the concepts of dialogue and negotiation are elementary, establishing that between two figures in which a relationship is established, in this case nursing professional and patient, there are inequalities and inequities. Thus, interculturality is considered one of the most sensitive trends to the needs for interaction, development, and growth of a culturally diverse population group that joins another that is culturally different.

A contribution that has been highlighted for the improvement of health care for migrants is Intercultural Health (*Salud Intercultural, SI*), which is an action recommended by various international organizations^(10,11,12)^. Intercultural health can be defined as “the interaction between cultures in a respectful, horizontal, and synergistic way, where the idea is that no cultural group is above the other, favoring at all times the integration and coexistence of both parties”^(13)^. It is inferred that those policies, programs, strategies, and actions that are developed under this concept shall consider the knowledge and practices in health, based on the individual or collective experience. It should be noted that it not only focuses on the interaction among people, but also on the need for integration and synergistic work of existing health systems, thus vindicating the historical evolution of populations that have been minimized or invisibilitized.

It allows delving into the cultural characteristics of individuals, families and communities and improving decisions about care, besides strengthening the relationship between nursing professionals and the patient, and the monitoring and adherence to the care plan, promoting elements of integration and trust in the community^(14)^.

In this regard, nursing science has developed a large number of theoretical and methodological proposals to achieve integration and respect in the care process, and to improve the way in which migrants are cared for, particularly in this case. Among these contributions, two proposals stand out: the Theory of diversity and universality of cultural care (Transcultural Nursing) and the Cultural Competence Model in caring for the person.

Initially, the Transcultural Nursing can be defined as:

“A formal area of humanistic and scientific knowledge and practice focused on the phenomena and competencies of holistic cultural care to help individuals or groups to maintain or regain their health (or well-being) and to cope with disabilities, death, or other human conditions, in a culturally coherent and beneficial way^(15)^. This contribution has gained strength over time thanks to the modification of socio-cultural spaces worldwide. Transcultural Nursing stands out for its generalization, because it recognizes culture as a global element of the human being, starting from the need to discover care and the individuals’ cultural behaviors, as a way of influencing their health, thus consolidating a consistent, safe, and responsible care activity^(16)^.

Moreover, every society develops forms of care specifically associated with its culture, for example, traditional Chinese medicine, or traditional Mexican medicine; however, if the person is not in their environment (in this case, the international migrant) their thoughts and forms of care are different, and barriers may arise, interfering with prevention, diagnosis, treatment, and recovery processes. Some barriers from the receiving systems are the lack of knowledge of the migrant’s language, the lack of knowledge about historical-cultural factors and intercultural health^(17)^. For the use of this theory, an understanding of concepts is required, as follows:

– **Cultural Care Diversity:** the differences in meanings, models, values, ways of life and symbols of care between societies or communities that are directly related to the application of care activities to the person (assistance, support, or training).
– **Universality of cultural care:** the similarity or uniformity in the meanings, models, values, ways of life, and symbols of care manifested among many cultures and that reflect care as an element of universal humanity.

This way, the theory states that in the relationship with the person, nursing staff shall provide culturally appropriate care, which can be considered as the “explicit use of care and culture-based health knowledge, in a sensitive, creative and meaningful way, that adapts to lifestyles and general needs of individuals or groups for well-being, and beneficial and satisfactory health or to cope with illness, disability or death^(15,18)^”. For this, the nursing staff should consider the application of one of these actions:

– **Cultural care conservation:** Actions and professional decisions of assistance, support, facilitation, and training that help people of a culture to recover or preserve significant care values, for their well-being, to recover from illnesses or to know how to face physical or mental impediments.
– **Adaptation of cultural care:** Those actions that allow the person to adapt or reach an agreement with other cultures, to obtain beneficial and satisfactory results.
– **Reorientation of cultural care:** They are actions that allow people to reorganize, change their life models (ways of caring) to obtain different and beneficial results.

They promote decision-making awareness of cultural needs, which reduces conflicts arising from health care due to cultural differences, which lead to distance between the health staff and the population, and prevents discrimination and the imposition of ethnocentric thinking^(19)^. This way, seeking a real approach to the person becomes fundamental, inviting us to understand the work methodologies to have an in-depth comprehension of the elements of life that are around health issues. Thus, the Cultural Competence in care emerges as a proposal to strengthen the contributions of the Transcultural Nursing theory.

On the other hand, one of the models that has stood out in the literature is the Cultural Competence Model (*MCC*). This model, provided by nursing, establishes the need to understand and know the influence of the culture of individuals, families, and communities in various aspects of daily life^(20–22)^, particularly in scenarios where health personnel, and especially nursing professionals, acquire an ethical and professional responsibility, in which understanding, knowing, and recognizing the value of people from their cultural contexts is vital within the process of care, which directly interferes with the nursing professional/person relationship^(21,23)^.

To make his proposal understandable, the author developed 20 principles that allow getting further in the ­methodological approach:

– One culture is not better than another; they are just different;
– There are similarities at the core of all cultures;
– There are differences within each culture and between them;
– Cultures change over time;
– If people are co-participants in care and have a choice to participate in health goals, plans and interventions, the expected results will be improved;
– Culture has a powerful influence on the interpretation of and responses to health care;
– Each individual has the right to be respected for their uniqueness and cultural heritage;
– Caregivers need general and culture-specific information to provide sensitive and competent care;
– Caregivers who can assess, plan, and intervene in a culturally competent way will provide better care for the people they care for;
– Prejudice and bias can be minimized with cultural understanding;
– Health care must reflect the unique understanding of the values, beliefs, attitudes, and world views of diverse populations for it to be effective;
– Every encounter with the patient is a cultural encounter.

Given these contributions, it is assumed that the greater the knowledge of the characteristics of the population studied, the greater the cultural competence. Thus, the model proposes four stages in this process:

– **Unconsciously incompetent:** stage in which the staff does not understand or know the person or population and therefore does not acknowledge importance to the provision of care based on a cultural view.
– **Consciously incompetent**: health personnel begin to get to know people incipiently and understand that it is necessary to delve into culture to provide culturally appropriate care.
– **Consciously competent:** Health personnel carry out actions to broaden their knowledge about the cultural elements of health and consciously reflect on the importance of cultural care.
– **Unconsciously competent:** It is when the health personnel is considered to know the person or population in depth and provide care based on a cultural approach.

Therefore, the model proposes a structure for the development of cultural competence and establishes its division into two areas: macro and micro. The macro focuses on the recognition of the nursing metaparadigm, which epistemologically supports the view of cultural competence^(20,21,24)^ and the micro, on the other hand, refers to the approach of 12 domains in which necessary elements to increase cultural competence are considered:

– General panorama, inhabited localities, and topography;
– Communications;
– Family roles and organization;
– Work issues;
– Biocultural ecology;
– High risk behaviors;
– Nutrition;
– Pregnancy and maternity practices;
– Practices and rituals associated with death;
– Spirituality and religious practices;
– Health care practices;
– Care providers.

With the use of these theoretical-methodological approaches, the development of care that is culturally congruent with culturally diverse populations is promoted, either in the development of community interventions or hospital approaches, thus promoting actions focused on personal, social, and cultural needs of these groups. Accordingly, the purpose of this study is to describe the construction process of an intercultural care program for international migrants in northwestern Mexico.

## METHOD

### Design of Study

It is the report of professional experiences, of a work group in a specific scenario and context that led to the construction of an intercultural care program aimed at international migrants in northwestern Mexico. This contribution is based on what was proposed by Daltro and Faria^(25)^, who propose the experience report as a relevant contribution that favors the production of scientific knowledge, since it recognizes the human condition and its complexity based on post-positivist paradigms. In this regard, compliance and development of the following methodological phases are established:

– To understand the experience report as a narrative.
– To consider that at least one author lives the phenomenon from real experience.
– To present the context, the actors and the ways of approaching the study phenomenon as it occurs in the real scenario precisely and objectively.
– To present a theoretical approach based on the literature that allows the construction of new knowledge.
– To narrate the experience in a clear and simple way, where the elements of the theoretical approach are reflected, without leaving out the possibilities of positioning, the generation of criticism, and reflection of the phenomenon.
– To avoid writing conclusions and propose considerations of results and lessons learned before, during and after the particular approach to the phenomenon.

### Population

The approach and work was carried with people in a situation of internal and particularly international mobility, all in the context of a city on the northwest border of Mexico in the period from 2018 to 2022.

### Sample Definition

A specific sample size was not established, while the sampling, in most cases, was by snowball, due to the conditions in which the phenomenon of international migration occurs, where flows and stays are irregular. The data regarding the number of people with whom the activities were carried out correspond to the particular records of each of them.

### Data Collection

The information of the experiences presented comes from the activities that have been developed in the Nursing School of the Universidad Autónoma de Baja California, (FEM-UABC), in the city of Mexicali, in the state of Baja California (BC) in Mexico, where efforts were made in 2018 and 2019 to provide prevention care. These activities were carried out in key community settings, such as points where migrants found temporary and low-paid jobs or near the border wall, also called “The Line” or “Trump’s Wall.”

### Data Analysis and Treatment

The data described is presented chronologically, in such a way that the progress and evolution of the actions carried out with the community of national and international migrants are demonstrated. As recommended, the narrative was made clearly considering the theoretical references and the positions for the development of reflections.

### Ethical Aspects

The presentation of this experience is not considered a research activity, therefore it was not submitted to the Research Ethics Committee of the academic entity. However, during the approaches to migrant communities, the bioethical principles and human rights of the people with whom interactions were established were followed.

## RESULTS

### Geopolitical Context

This contribution is contextualized within the northwest border landmark of Mexico-United States, in the city of Mexicali, Baja California (BC), Mexico, which is a point where people of different nationalities arrive through land or air and it is also a regular and irregular point of entry to the State of California in the United States.

From January to December 2022, the migration statistics of the Government of Mexico, regarding people in an irregular situation in BC, refers to the presence of 44.411 people, of which 9.097 were located in this border city. In this scenario, international migrants in a regular situation with temporary (299) and permanent (649 people) visas, as well as those who have requested a stay for humanitarian reasons (251), must also be considered. This situation implies the presence of people from different contexts and socio-cultural needs^(26)^. A large part of this population of migrants is subject to the regulations of the immigration policy of both Mexico and the United States, since many are in the Mexican territory, waiting to receive a response to their asylum claim according to the United States guidelines and policies.

When relating this situation to health care, the fact that Mexico is a country that establishes universality in health, which grants every person who is in the Mexican territory the right to access its protection, is highlighted, and that is why the Mexican government developed the Comprehensive Health Care Plan for the Migrant Population^(27)^ which proposes a structure that allows migrants to access medical care, health surveillance, and public health services, responding to the wide range of health problems. Initially, health promotion was carried out on topics such as prevention of hypertension, diet, prevention of addictions and sexually transmitted infections, in addition to anthropometric measurements (weight and height).

In these approaches, which included health brigades, humanitarian aid campaigns for clothing and food, and research projects on community intervention, work was done with migrants from Honduras, El Salvador, Guatemala, and Haiti, as well as those from the south of Mexico. These actions were considered to contribute to those established in the Comprehensive Health Care Plan for the Migrant Population (*PIASPM*) of the Mexican government. However, at various times when professors and researchers sought the collaboration of public health institutions that provided care to vulnerable populations, it was highlighted that the efforts made by them were not structured according to the *PIASPM* and only responded to emerging social and health needs.

By the year 2020, with the start of the COVID-19 pandemic, academic activities were restricted; however, the reality and importance of the migratory phenomenon in Mexico increased, a situation that brought the “Migrant Caravans” that left Central American countries heading to the various cities on the northern border of Mexico. Therefore, FEM-UABC academics, students, graduates, and civil associations continued with activities to prevent the transmission of COVID-19 and the prevention of Sexually Transmitted Infections (STIs); within the most representative populations, migrants of Central American origin and Haitians who resided in vulnerable areas of the city are highlighted.

The cultural differences became evident with the Haitian population (mainly language) and established the need to address disease prevention from an intercultural approach, a situation with which actions and studies were developed to strengthen cultural competence in what regards access to health services during their transit through the northwestern region of Mexico and on the topic of STI prevention. This situation led to meetings with social and spiritual leaders (barbershops and churches) of the community and later to coexistence and routine interaction.

In the process of knowledge of cultural constructs, the Cultural Competence model was used, where through meetings, interviews, and interaction with the various communities, there was a progression to stage number three (consciously competent), which allowed understanding the functioning and representation of health in its migration context. In view of this, maintaining health status becomes critical to allow people to go on moving, while accidents and injuries hinder continuing on the road. The perceived difficulties in accessing health services due to the language were also marked, where the effort of local personnel was focused on explaining with signs and from Spanish, the actions to be carried out. The perception of discrimination based on skin color is also taken up again, a situation associated with long waits in health institutions.

The increase in the demand of care services for Haitian women’s pregnancy, childbirth, and puerperium, as well as the strengthening of relations between Haitian men and Mexican women, were elements of great interest generated from the observation in the field work. In this regard, and from the perspective of the population addressed, the use of *“kapot”* (Haitian Creole word for condom) is out of step with religious beliefs. As a result of these interactions, significant and sensitive learning was obtained in terms of education for condom use and from which qualitative articles were published that allowed increasing cultural competence^(28,29)^, leading to the comprehension of the importance of religious beliefs in protected sexual relations and the perception of access to health services during transit to northern Mexico.

As a result of all this, in the year 2021, the cultural adaptation of an intervention was carried out to increase the use of condoms by Haitian migrant men, in which beliefs, language, habits, and even social and labor dynamics were considered in the Mexican context. Two courses of three sessions each were given, with a total population of 34 migrant men. The activities addressed topics about STIs and HIV, myths and realities, training for the use of the external condom and considerations regarding sexual health during the migration process. This way, a close relationship with the Haitian migrant community was consolidated, which allowed continuing with health activities, as well as volunteer service.

The experience regarding the approach of the diverse populations over the years led a member of the group of FEM-UABC’s professors to pursue specialization studies on international migration during the year 2022, in which he proposed, as a final project, the evaluation of the Mexican program established for the migrants health care. For this, meetings were held with those responsible for shelters and public humanitarian support centers to understand the scope of the public health system, as well as their real needs, a situation that led to meetings with OIM representatives and efforts to donate food and winter clothing, and the development of health brigades to shelters in vulnerable areas of the city of Mexicali.

With all the experience acquired over the years and with the increase in cultural competence regarding health needs and the phenomenon of international migration, the processes began to consolidate a proposal that would allow an approach by organized groups of university students, for which a Community Social Service Project called Health Program for Migrants, with an Intercultural Approach “*Salud Migrante*” (Migrant Health in English), was designed and created, the objective of which was to develop activities for disease prevention and health promotion with an intercultural approach, in shelters, refuges, and temporary camps in the city of Mexicali Baja California, Mexico.

The structure of this project implies that students are trained in international migration, current migratory flows in the region of the Americas, intercultural health, and cultural competence. Thus, the proposal is to develop, in each institution, nine sessions of approximately one hour with educational strategies focused on the community, under the following thematic axes: tuberculosis, diabetes mellitus, arterial hypertension, gastrointestinal infections, oral health problems, addiction prevention, prevention of gender and/or sexual violence, cancer prevention in women and men, prevention of HIV and other STIs, and promotion of maternal and child health.

The application of the *Salud Migrante* project during 2023 aims to generate a social impact, to promote access to disease prevention and health promotion services for national and international migrant populations, at risk of developing or acquiring communicable and noncommunicable diseases, alterations caused by environmental and animal factors, injuries due to violence and accidents, in the context of the northwest border of Mexico.

To date, the *Salud Migrante* program has been selected and approved by internal calls of this university and is registered as active in the Comprehensive System of Social Service of the UABC. It receives financial resources for student scholarships, with students from all areas of knowledge, particularly from the area of health, being invited to contribute with actions or strategies from their disciplines to improve the health of the international migrant population. In the long term, the creation of groups of nursing students for the migrants’ health is expected, in the Mexican cities through which they transit to the United States.

## DISCUSSION

The construction of health-related proposals, based on the work with migrant populations, is an issue that allows demonstrating the need to incorporate intercultural health and cultural competence in nursing care, which strengthens the provision of congruent cultural care. A globalized world that favors migratory movements requires a great commitment from public and private health services. Health professionals have acquired the responsibility of providing quality care based on scientific and humanistic knowledge, developing community-type projects, promoting quality care, and adding efforts to international recommendations for health universality, a situation in which the migrant population, at least in the context of Latin America and the Caribbean, has greater opportunities to achieve the migratory objectives of better health conditions.

The challenge of applying this knowledge in structured programs is vast, since it implies, to a great extent, personal dedication and large economic financing to effectively influence the complex scenarios in which migrants find themselves. Moreover, the permanent link with State agencies becomes evident, for the coordination of efforts and jointly to be able to comply with the application of national and international laws, treaties and agreements.

The northwest border of Mexico has been characterized by the reception of people from China, Haiti, Venezuela, Honduras, Guatemala, Brazil, Cuba, etc., and even, and derived from the current geopolitical situation, from Ukraine and African countries; all these people have unique elements that make them stand out from the rest; therefore, nursing staff must consider the cultural factors that constitute them in a holistic approach, promoting a science of care that is more sensitive to the needs of people.

## CONCLUSION

The experience in the process of caring for migrant populations has witnessed the need to strengthen actions from intercultural health, something that implies the development of cultural competencies by the nurses who are in contact with this population. The use of cultural care theories and models promotes a more sensitive approach to the needs of international migrants. Care actions can be extended during the various points of transit, which implies coordination between nursing personnel from health institutions in various countries, as well as from higher-level academic institutions, to help these people maintain an optimal health status before and during the journey to the destination country.
